# A publication-wide association study (PWAS), historical language models to prioritise novel therapeutic drug targets

**DOI:** 10.1038/s41598-023-35597-4

**Published:** 2023-05-24

**Authors:** David Narganes-Carlón, Daniel J. Crowther, Ewan R. Pearson

**Affiliations:** 1 Division of Population Health and Genomics, Ninewells Hospital, School of Medicine, University of Dundee, Dundee, DD1 9SY UK; 2 Exscientia Ltd, Dundee One, River Court, 5 West Victoria Dock Road, Dundee, DD1 3JT UK

**Keywords:** Target identification, Target validation

## Abstract

Most biomedical knowledge is published as text, making it challenging to analyse using traditional statistical methods. In contrast, machine-interpretable data primarily comes from structured property databases, which represent only a fraction of the knowledge present in the biomedical literature. Crucial insights and inferences can be drawn from these publications by the scientific community. We trained language models on literature from different time periods to evaluate their ranking of prospective gene-disease associations and protein–protein interactions. Using 28 distinct historical text corpora of abstracts published between 1995 and 2022, we trained independent Word2Vec models to prioritise associations that were likely to be reported in future years. This study demonstrates that biomedical knowledge can be encoded as word embeddings without the need for human labelling or supervision. Language models effectively capture drug discovery concepts such as clinical tractability, disease associations, and biochemical pathways. Additionally, these models can prioritise hypotheses years before their initial reporting. Our findings underscore the potential for extracting yet-to-be-discovered relationships through data-driven approaches, leading to generalised biomedical literature mining for potential therapeutic drug targets. The Publication-Wide Association Study (PWAS) enables the prioritisation of under-explored targets and provides a scalable system for accelerating early-stage target ranking, irrespective of the specific disease of interest.

## Introduction

Numerous machine learning strategies have demonstrated the potential to accelerate drug discovery across various stages, such as drug repurposing, de novo drug design, clinical trial optimisation, and patient selection^1,2^. Target identification and prioritisation represent critical initial steps in the drug discovery process. Historically, only 10% of drug targets successfully progress through clinical trials^3,4^, and the success rate appears even lower for novel targets^5,6^. Traditionally, individual researchers have relied on their expertise to analyse scientific literature and prioritise potential opportunities. Recently, numerous efforts have employed machine learning techniques to automate and prioritise gene-disease associations^7–13^. However, most of these studies have focused on training models and validating them on pre-existing therapeutic targets, rather than adopting a time-sliced approach to train on past data and evaluate the models' prospective predictions. Additionally, these approaches primarily rely on structured property databases as their main source of machine-interpretable data, which only encompass a limited portion of the knowledge found in the research literature. Paliwal et al. utilised a knowledge graph approach combined with link prediction methods to historically prioritise targets, claiming that their method successfully identified 1 in 4 therapeutic relationships that were eventually proven true.

Scientific publications contain valuable relationships and inferences that the research community can interpret, with PubMed housing over 35 million publications as of 2023. The volume of scientific literature will only continue to grow, creating a vast repository of undiscovered public knowledge. The biomedical domain has seen a surge of interest in pre-trained language models, owing to their remarkable success in the general natural language domain. The pre-trained models can be classified into two main categories: BERT and GPT, along with their respective variants. BERT covers models like BioBERT^14^ and PubMedBERT^15^, whereas GPT includes BioGPT^16^. These transformer language models have been pre-trained on biomedical literature. Typically, these models are evaluated and benchmarked on tasks such as entity recognition, relationship extraction, and question answering for known biomedical applications, following their training on the entirety of the biomedical literature. However, assessing their performance on genome-wide prioritisation of therapeutic targets presents a challenge, as these models often divide disease and gene synonyms into multiple tokens, complicating the process of consolidating them into a cohesive ranking.

In this study, we introduce a Publication-Wide Association Study (PWAS) to assess the ability of independently trained models using historical data to prioritise unstated hypotheses, which could be considered false positives or ‘hallucinations’^17^, and are eventually validated. Additionally, we examine the potential of historical Word2Vec models to prioritise yet-unknown hypotheses by utilising future test sets instead of relying on test datasets containing already established associations that the language models have already seen. By training unsupervised language models on historical subsets of biomedical literature, we explore whether these models could have effectively prioritised or suggested novel drug discovery scientific hypotheses in the past. To this end, we evaluate the language models on a range of drug discovery-related tasks, including target classification, gene-disease prioritisation, and protein–protein interaction prediction, within the context of a historical, retrospective study.

Protein–protein interactions are also crucial in drug discovery because data incompleteness in the human interactome hinders our understanding of the molecular roots of human disease^18^. Drug targets with genetic evidence are twice as likely to succeed in clinical trials^19,20^. However, most immune-related targets are found in the protein–protein interaction vicinity of genetically associated genes rather than being genetically associated themselves^21^. Therefore, it is essential to understand systems biology to propose novel targets. Moreover, validating protein–protein interactions can be time-consuming and costly, but computational tools offer a cost-effective alternative to prioritise novel protein–protein interactions^18^ and suggest new therapeutic targets. There have been numerous methods to predict protein–protein interactions using a mixture of traditional machine learning methods^22^ and deep learning models^23–25^ that include AlfaFold2 protein structure information. However, this work focuses on whether language models could have prioritised novel interactions with the limited data available in the past scientific literature.

In this work, we introduce a Publication-Wide Association Study (PWAS) that examines whether language models can retrospectively prioritise gene-disease associations before their initial statements, target-disease indications before the conception or synthesis of drug-like molecules, and protein–protein interactions before their discovery.

## Results

### Target-disease prioritisation

**Figure 1:**
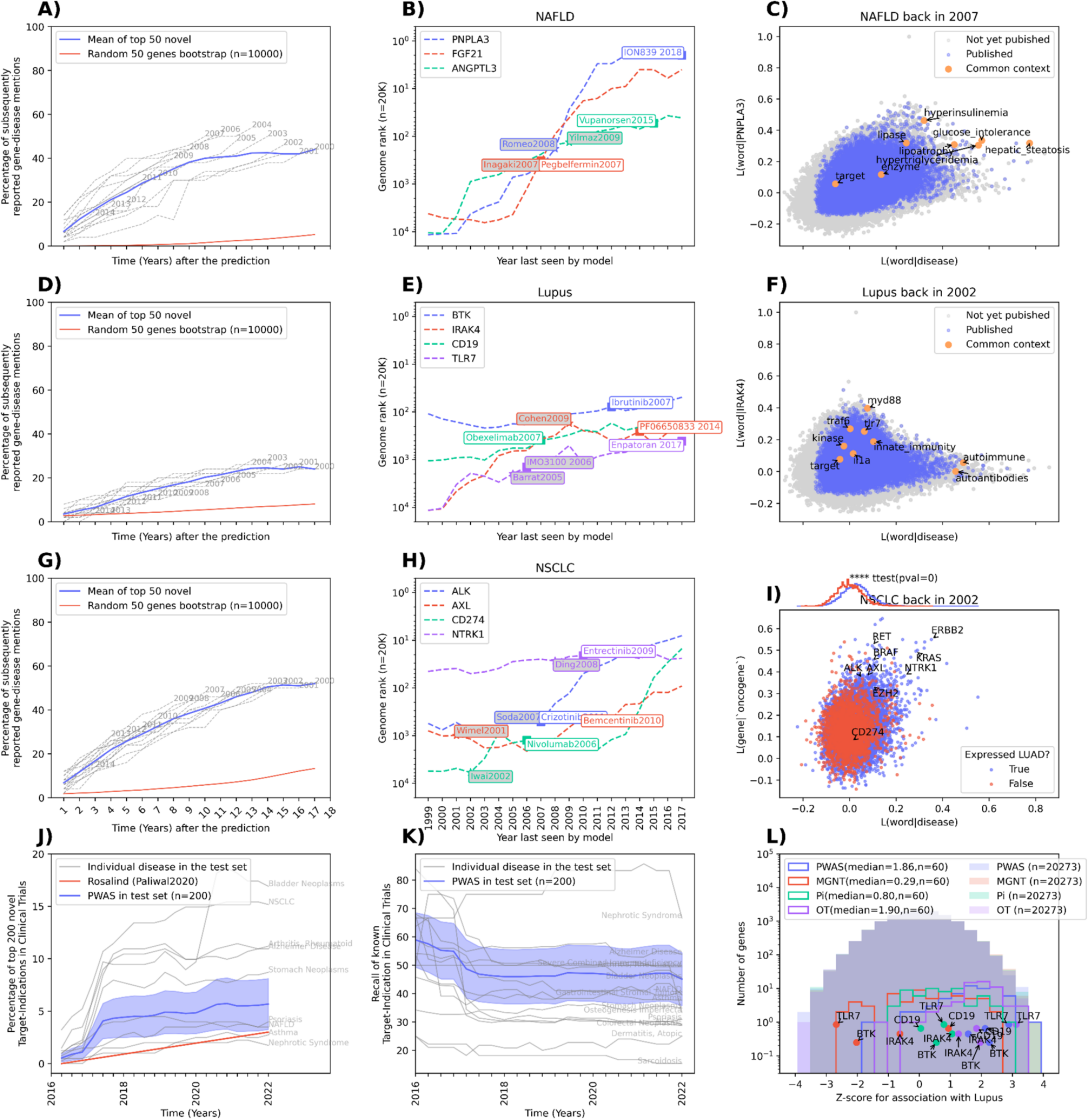
(**A**,**D**,**G**) Prospective gene-disease association predictions based on historical datasets. Grey lines represent the cumulative percentage of predicted genes that were subsequently reported in the years following their predictions using abstracts published before that year; earlier predictions span longer testing periods. The results are averaged (blue) and compared to a random bootstrap sampling of 50 genes in 10,000 iterations. (**A**) for non-alcoholic fatty liver disease (NAFLD), D) for systemic lupus erythematosus (SLE), and (**G**) for non-small cell lung carcinoma (NSCLC). (**B**,**E**,**H**) Historical gene ranking scores. Each subplot displays genome-wide ranking scores (n = 19,229) from 1999 to 2017. The first author's name and publication year are presented in the coloured, grey-filled square adjacent to the ranking plot. The drug name and the year of first reported evidence are displayed in the coloured, white-filled square adjacent to the ranking plot. (**B**) for NAFLD, (**E**) for SLE, and (**H**) for NSCLC. (**C**,**F**,**I**) Likelihood plots for interpretability. (**I**) Illustrates the likelihood of some oncogene targets of NSCLC co-occurring with NSCLC (x-axis) and the word ‘oncogene’ (y-axis), coloured by mean gene expression levels from The Cancer Genome Atlas (TCGA). Rankings are enriched (t-test with p-value = 1e−189) for genes expressed in lung adenocarcinoma cells (threshold 0.1 transcripts per million, TPM). (**J**) Subsequent target-disease links entering trials. Grey lines indicate the cumulative percentage of top-scoring target-indication pairs reported in clinical trials following their 2016 predictions. Each line represents a unique disease in the test set (n = 200). The results are averaged (blue with standard deviation). Metrics for the 5-year window prediction from 2015, as described by Paliwal et al.^12^ Rosalind method, are plotted in red. (**K**) Prospective recall at 200. As more target-indication hypotheses are tested, recall decreases (**B**) over the subsequent years (x-axis) as more target-indication pairs enter clinical trials. The recall for 25 of the 200 diseases in the test set is plotted with their names in grey, and the mean for the 200 diseases is plotted in blue. (**L**) Target z-scoring for SLE. Four rankings are compared: PWAS (ours), MAGENTA (MGNT), Pi (Priority Index), and Open Targets (OT). Outer-shaded histograms display the normalised z-scores for 19,229 human protein-coding genes. Inner histograms present normalised z-scores for 60 SLE targets with FDA-approved drugs or in clinical trials.

**Figure 2:**
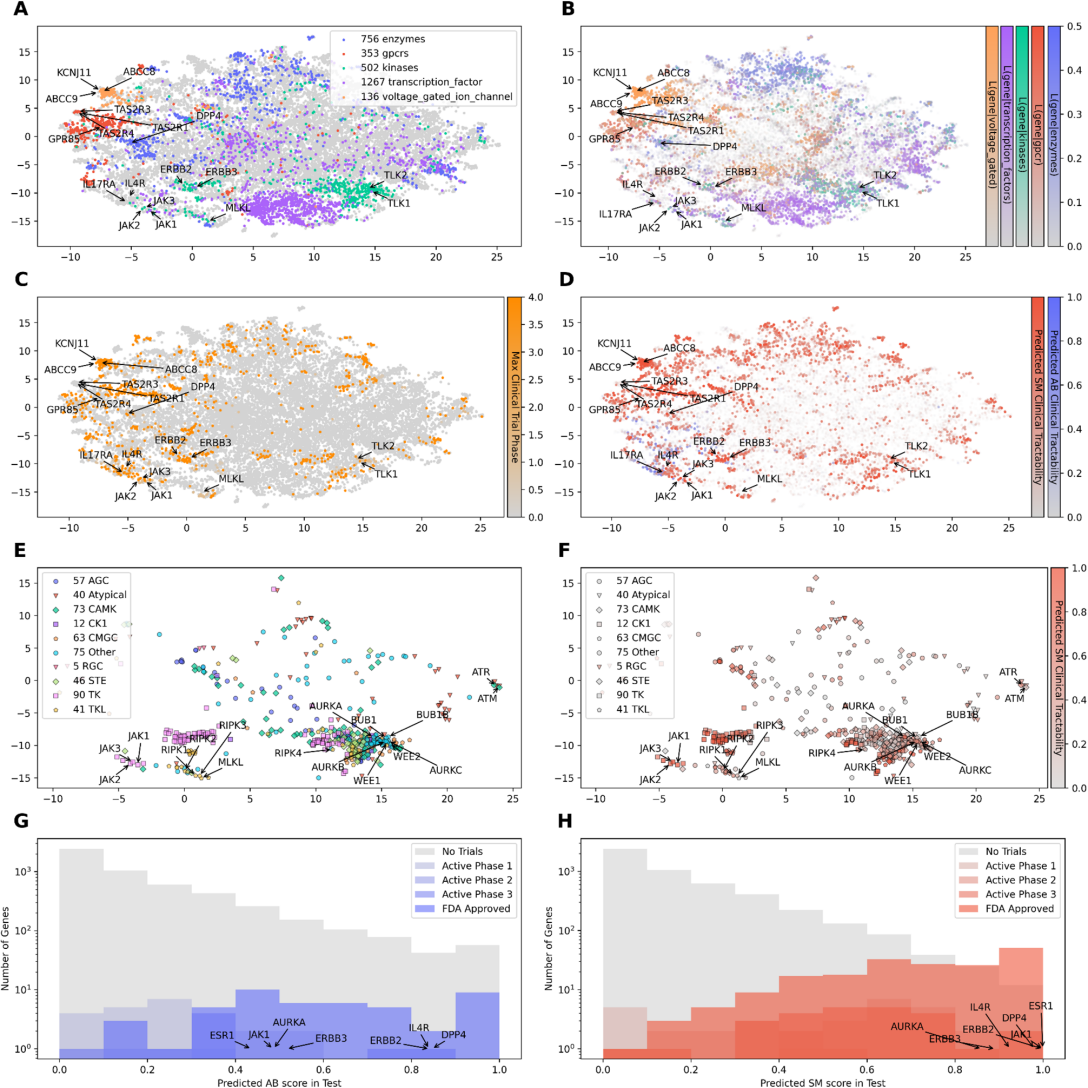
Uniform Manifold Approximation and Projection (UMAP) visualisation of word2vec embeddings for the published human protein-coding genome (n = 19,229 data points). (**A**) The first two UMAP components depict a lower-dimensional representation that visualises several therapeutic drug target classes, including enzymes, G-protein-coupled receptors (GPCRs), ion channels, and kinases. Transcription factors, previously deemed undruggable, are also shown, with their druggability enabled by the advent of PROTACs. (**B**) The UMAP lower-dimensional representation is coloured according to the word co-occurrence likelihoods of gene tokens and several tokens related to the therapeutic target classes (enzyme, GPCRs, voltage-gated, kinases, transcription_factors), emphasising the same areas as subplot (**A**). (**C**) The same UMAP representation is now coloured based on the maximum clinical trial phase achieved, as documented in PharmaProjects. (**D**) Predicted small molecule (SM) and monoclonal antibody (AB) tractability estimates are obtained using a multi-task logistic regression classifier, which uses human gene embeddings as input. These gene embeddings can be considered fingerprints for downstream estimation tasks. (**E**) A subset of genes encoding human kinases is displayed, with colours denoting different kinase families: AGC for kinase A, G, and C families (PKA, PKC, PKG); CAMK for calmodulin-dependent protein kinase; CK1 for casein kinases; CMGC for cyclin-dependent kinases, mitogen-activated protein kinases, glycogen synthase kinases, and CDK-like kinases; RGC for receptor guanylate cyclases; STE for serine/threonine kinases; TK for tyrosine kinase; and TKL for tyrosine kinase-like. (**F**) The same plot as in (**E**) is presented, but with colouring based on the predicted clinical tractability of small molecules.

We trained multiple Word2Vec^26^ language models on an English corpus of 19.5 million biomedical titles and abstracts to generate embeddings for a vocabulary of approximately 2 million phrases (see “Methods” for details). Language models were saved at the end of each year to enable retrospective analysis (see “Methods” for details). Language models leverage co-occurrence information from a text corpus to estimate the likelihood of two words or phrases co-occurring, even if they have never appeared together in the training set. These likelihood scores can be employed to rank gene-disease hypotheses that have not been explicitly stated before. We trained language models on literature up to various points in the past and subsequently evaluated their ranking on prospective gene-disease associations published in the literature. Specifically, we generated 28 distinct historical text corpora consisting of abstracts published between 1995 and 2022, with each incrementing the cut-off date by one year. Independent models were trained on these historical datasets and used to prioritise associations between genes and diseases that were likely to be reported in future (test) years (Figs. 1A, 2D,G).

For each year, the language models were used to make a prediction. We tested which percentage of the top-ranked genes without prior publications were subsequently reported in the literature (Fig. 1A,D,G for NAFLD, SLE and NSCLC). The grey lines correspond to the predictions made by a different language model trained with one of the 28 different historical datasets. For example, the grey line labelled ‘2012’ in Fig. 1A depicts the percentage of the top 50 novel predictions from the 2012 model that were found to be associated with the following years. The cumulative precisions are averaged (red line in Fig. 1A,D,G) and compared to random (bootstrap sampling of 50 genes without replacement in 10,000 iterations). Approximately 50% of the top 50 novel genes are reported to be associated with diseases in different therapeutic areas (metabolic disorders, immune disease and oncology) after several years (Fig. 1A,D,G for non-alcoholic fatty liver disease (NAFLD), systemic lupus erythematosus (SLE) and non-small cell lung carcinoma (NSCLC)). Overall, the language model predictions (blue lines in Fig. 1A,D,G) were 6 to 10 times more likely to be studied than a random sample of genes (red in Fig. 1A,D,G).

The genome-wide rankings from the language models were then evaluated on prioritising therapeutic drug targets before their discovery in a scientific publication and the first clinical trial in Fig. 1B,E,H for NAFLD, SLE and NSCLC, respectively. The genome-wide rankings for the human protein-coding genes PNPLA3, FGF21, and ANGPTL3 (Fig. 1B) rapidly increased before the first publications relating them to NAFLD: PNPLA3 by Romeo et al.^27^ in 2008 and FGF21 by Inagaki et al.^28^ and ANGPTL3 by Yilmaz et al.^29^ in 2007 (Fig. 1B). Furthermore, the genome-wide rankings rapidly prioritised these targets before the first clinical trial with compounds modulating their activity: ION-839l to inhibit the production of PNPLA3 in 2018, pegbelfermin as a human FGF21 analogue, first reported in 2007^30^, Vupanorsen as an *N*-acetyl galactosamine-conjugated antisense molecule that targets ANGPTL3 mRNA in 2015^31^ (Fig. 1B). Language models are sometimes black boxes, but we gained insights into what drove the predictions by finding ‘contextual linking words’ already published and then prioritising articles containing them. Figure 1C shows the intermediate ‘contextual linking words’ for PNPLA3 and NAFLD. These words include hyperinsulinemia, glucose intolerance, hypertriglyceridemia as terms related to the claims from research performed from 2004 until 2007, that associated PNPLA3 with obesity, insulin resistance in adipose tissues^32–34^, the phenotypes of NAFLD.

The same applies to the immune-related trait SLE in Fig. 1E. Bruton tyrosine kinase (BTK) ranked in the top-100 genes in 1999, well before the trial of ibrutinib in 2007^35^. CD19 ranked in the top-300 genes before the first trial of obexelimab in 2007^36^. Both interleukin-1 receptor-associated kinase 4 (IRAK4) and Toll-like receptor 7 (TLR7) were ranked from the top-10000 to the top-1000 genes for SLE before the first publication implicating them in the pathology of lupus, in 2009 by Cohen^37^ and 2005 by Barrat et al.^38^, respectively. IRAK4 was consistently ranked in the top-1000 genes prior to the first PF06650833 inhibitor by Pfizer which dates back to 2014^39^. Similarly, TLR7 was on the top ranking for several years before the first TLR7 inhibitor that entered the clinic, IMO3100, which was discontinued. Enpatoran is another TLR7 inhibitor currently in Phase 2 trials for lupus^40^. The contextual linking words indicate why the model suggested IRAK4 for lupus, due to its involvement in innate immunity through TLR7 that subsequently activates Myd88^41,42^ (Fig. 1F).

Figure 1G–I are related to NSCLC. Figure 1H shows that anaplastic lymphoma kinase (ALK) and AXL receptor tyrosine kinase ranked in the top 1000 suggested targets for NSCLC for several years before their first evidence by Soda et al.^43^ in 2007 and Wimmel et al.^44^ in 2001, and the first trials for the ALK inhibitor crizotinib (originally thought as a tyrosine-protein kinase MET specific inhibitor) in 2007^45^ and the AXL inhibitor bemcetinib in 2010^46^. The 2006 language model was aware of their role as receptor tyrosine kinase oncogenes (high similarity to ‘kinase’ and ‘target’ Fig. 1I) in other cancer indications^47,48^ and their potential implication in NSCLC. The immunomodulatory role of CD274 in tumours was demonstrated by Honjo’s group, winner of the Nobel Prize in 2002^49^. The language model could not prioritise this gene in the top 1000, but it did before the first monoclonal antibody nivolumab, in 2006^50^. There was prior evidence of the neurotrophic receptor tyrosine kinase 1 (NTRK1) association with types of NSCLC in 1995^51^ but the oncogenic role in lung adenocarcinoma was not confirmed until 2008 by Ding et al.^51^. NRTK1 ranking was consistently in the top 100 genes (Fig. 1H) before Ding results and in the top 20 (Fig. 1H) before the first evidence of an NRTK inhibitor, entrecitibib, in 2009. Furthermore, back in 2002, language models were able to prioritise several oncogene targets of NSCLC, genes expressed in lung adenocarcinoma (LUAD, Fig. 1I) cell lines (see “Methods”, t-test with p-value 1e − 198) but not CD274. This suggests that the top-ranking targets are enriched in genes expressed in the tissue of origin of the disease.

The embeddings from 2016 were used as covariate features to estimate the clinical trial success (see “Methods”) with a multilayer perceptron model (see “Methods”). The output generated another score that was tested on prioritising incipient target-disease indications that ended up in clinical trials. On a testing set of 200 diseases described by Paliwal et al.^12^, 5.81% of the top 200 targets per disease novel suggestions ended up in a clinical trial after 5 years (Fig. 1J). These results outperform Paliwal et al.^12^ method with 3.54% precision for 200 targets per disease (Fig. 1 top 100a test to compare the performance of PWAS to other genome-wide ranking methods like MAGENTA^52^ (MGNT in red with Wang et al. 2021 data^53^ in Fig. 1L), Priority index^21^ (Pi in green Fig. 1L), Open Targets (OT in purple in Fig. 1L), we found that PWAS prioritises better (mean z-score is 1.86 in Fig. 1L) the 60 targets of SLE that have approved drugs or drugs in active clinical trials in October 2022 in Pharmaprojects^54^ (see “Methods”). Furthermore, it is the method that gave the highest z-score to the therapeutic targets TLR7, BTK and IRAK4 of which only TLR7 has been reported to have a genetic association with lupus^55^ through a single gain-of-function mutation^56^. These results suggest that language models prioritise gene-disease associations before their discovery and validation in clinical trials.

### Therapeutic target representation

An in-depth exploratory analysis was conducted using the 2022 model to investigate how human genes, as a biomedical entity, were represented in a low-dimensional space. Employing Uniform Manifold Approximation and Projection (UMAP), we uncovered hidden structures within the embeddings in this lower-dimensional space (Fig. 2A–F). Distinct therapeutic drug target families were found to cluster in separate regions of this space (Fig. 2A). The mean Silhouette Coefficient, which measures the consistency of gene families in the lower-dimensional space, was calculated to be 0.11 (with a maximum possible value of 1).

The mean cosine distance, a metric used to measure the similarity between two vectors or embeddings, was calculated for different gene families: 0.14 for all human genes, 0.19 for enzymes, 0.28 for G-protein coupled receptors (GPCRs), 0.27 for kinases, 0.23 for transcription factors, and 0.38 for voltage-gated ion channels (see “Methods” for details). The gene family clusters observed in Fig. 2A overlap when human genes are coloured based on the likelihood of co-occurring with phrases describing the respective gene families (Fig. 2B).

Focusing on individual genes, the gene embeddings clustered voltage-gated ion channel genes, such as ABCC8, ABCC9, and KCNJ11, which are targets for type 2 diabetes mellitus; JAK1, JAK2, and JAK3 kinases, which are therapeutic targets in numerous immune-related disorders; and receptor tyrosine kinases ERBB2 and ERBB3. Moreover, therapeutic drug targets with programs in clinical trials or with approved drugs were predominantly located in the periphery of the UMAP plot (Fig. 2C). These vector embeddings encapsulate information from the literature as dense fingerprints, which can be utilised in subsequent classification tasks.

To assess whether the embeddings contained information regarding successful clinical trial targets, the classification experiment conducted by Ferrero et al.^9^ in 2017 was replicated. Ferrero et al.^9^ utilised several manually engineered features from the Open Targets^13^ platform to predict whether a gene was a therapeutic target or not. In this study, the features employed were the word vector embeddings trained until December 2017 (see “Methods”). A multilayer perceptron with default parameters and a balanced loss function was used as the model. Genes were labelled as targets if Pharmaprojects^54^ tagged them in any active clinical trial or as registered or launched in 2017, following the approach employed by Ferrero et al.^9^. For targets with programs spanning multiple phases, the most advanced stage was considered. The choice of 2017 allowed for a direct comparison with the state of literature and clinical trial phases at the time when Ferrero et al.^9^ conducted their research.

Table 1 presents the classification metrics for the testing set with unseen genes relative to a random forest classifier. These metrics were calculated using a comparable number of negatives and positives. Precision represents the fraction of actual targets relative to all predicted targets, while recall is the fraction of predicted targets compared to the number of test targets. The f1 score, a metric that averages precision and recall, was found to be 20% higher than that reported by Ferrero et al.^9^ in the Approved or Clinical Trial column. Open Targets integrates manually engineered variables from multiple sources^13^ to define a gene as a target. These findings suggest that word vectors capture more information about therapeutic targets than expert-crafted variables. Classifiers could potentially be employed to generate a learned clinical tractability estimate.

**Table 1:** Evaluation of classification metrics on a balanced test set. The classification metrics for different classes are derived from Pharmaprojects^54^ and are assessed on a balanced test set containing an equal number of positive and negative samples. The values enclosed in brackets represent the 5% and 95% confidence intervals, which were determined by conducting 100 bootstrap sampling experiments, each maintaining an equal distribution of positive and negative samples. This approach provides a more robust estimation of the model's performance and ensures that the observed results are not influenced by any potential sampling bias.

Classification metric	Target in clinical trial	Target has an approved drug	Approved or clinical trial
Accuracy score	0.719 [0.665, 0.849]	0.801 [0.76, 0.841]	0.877 [0.822, 0.908]
F1 score	0.618 [0.558, 0.673]	0.667 [0.602, 0.697]	0.875 [0.808, 0.993]
Precision	0.498 [0.431, 0.531]	0.543 [0.467, 0.574]	0.889 [0.838, 0.932]
Recall	0.816 [0.712, 0.848]	0.864 [0.792, 0.917]	0.862 [0.829, 0.938]

The classification experiment was repeated separately for small molecule (SM) and monoclonal antibody (AB) targets (Fig. 2G). Figure 2D illustrates the predicted SM clinical tractability probabilities for the entire genome, while Fig. 2F displays probabilities for all human kinase genes. Despite the absence of monoclonal antibodies against known type-2 diabetes small molecule targets ABCC8, ABCC9, DPP4, and KCNJ11, these genes demonstrated AB tractability estimates greater than 60%, along with interleukin receptors IL4R and IL17RA (Fig. 2D). As transmembrane proteins, these genes are amenable to monoclonal antibody targeting, and the language model accurately captured this information despite the lack of evidence in the training set. Similar results were observed for ERBB2 and ERBB3, with 90% SM and AB scores, as well as FDA-approved small molecules and antibodies against them. Figure 2E contains only the 502 kinases (green Fig. 2A representing all 16,533 genes) represented in the lower dimension. In Fig. 2E,F, genes encoding protein kinases cluster based on their biological pathways and functions. Receptor-interacting serine/threonine-protein kinases RIPK1, RIPK2, RIPK3, and MLKL involved in necroptosis, as well as Janus tyrosine kinases JAK1, JAK2, and JAK3, cluster at the bottom centre of Fig. 2E. Additionally, kinases involved in DNA damage repair cluster together in Fig. 2E. Regarding small molecule tractability estimates, MLKL, TLK1, and TLK2 receive very low scores, as they are kinase-like proteins that have lost their kinase activity. The kinase families in the legend are the acronyms used by Guide to Pharmacology database^57^. These results suggest that language embeddings contain features valuable for defining therapeutic drug targets. However, the critical step in drug discovery is identifying the appropriate therapeutic drug target for a specific disease.

### Protein interactions

The limitations of the current human interactome pose challenges to comprehending the molecular underpinnings of human diseases^58–60^. For identifying potential therapeutic targets, it is essential to understand the pathways impacted by the disease and pinpoint nodes within the signalling network whose modulation can alleviate the disease state^58–60^. We investigated if language models prioritise protein–protein interactions (PPIs) before they are published. The historical data of PPIs were obtained from yeast-two-hybrid assays of the human interactome^58–60^, which consists of four datasets: H-I-05 from 2005, HI-II-14 from 2014, HuRI from 2020, and HI-union from 2020. A bona fide negative set was absent. Combinations of 'sticky' proteins, representing the top 10% of proteins with the highest number of interactions, served as negative examples, except when they overlapped with the positive set (refer to “Methods”).

**Figure 3:**
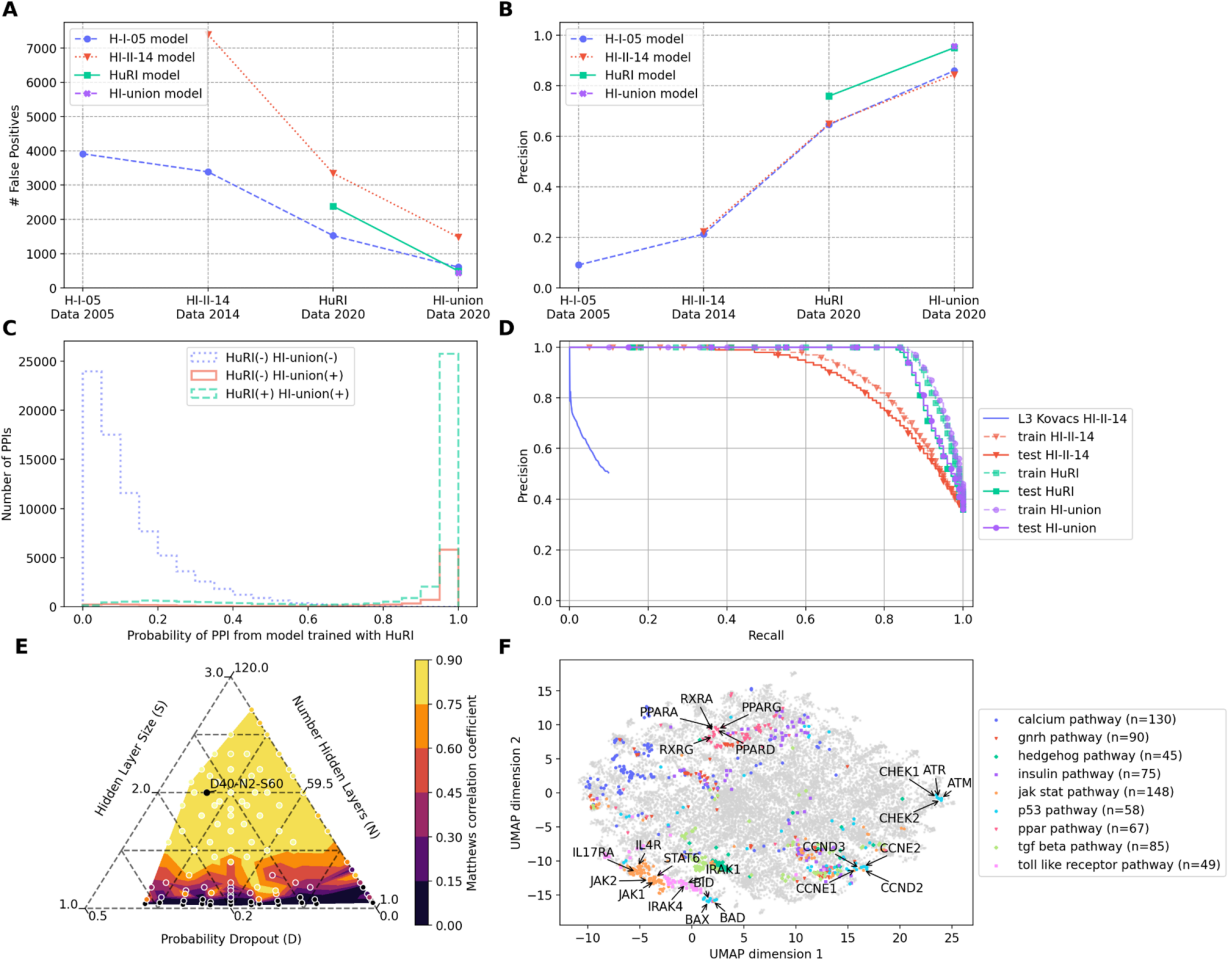
(**A**,**B**) Subplots display the historical prediction accuracies for the Human Interactome^58–60^. Subplot (**A**) presents the number of false positives, while subplot (**B**) illustrates the precision over time for different historical datasets (H-I-05 in blue, HI-II-14 in red, HuRI in green, and HI-union in purple), focusing exclusively on the testing set. Earlier models are assessed on newer datasets; for instance, the H-I-05 model was trained on H-I-05 positive examples and evaluated on subsequent datasets. (**C**) A histogram of predicted scores for positive, not-yet-positive, and negative sets of HuRI and HI-union is shown. The histogram plot displays the scores from the model trained on HuRI positive examples, with the x-axis representing the HuRI examples and the y-axis representing the number of protein–protein interactions. HuRI negatives are depicted in blue, positives in red, and HuRI negatives with HI-union positives in green. The HuRI model achieved an area-under-the-curve (AUC) score of 86.6% on the HuRI dataset and 89.7% on the HI-union dataset. (**D**) PWAS consistently outperforms the L3 method from Kovacs et al.^18^ (mean cross-validation in blue). 25% of the genes and all their interactions were used in the test set, while the remaining interactions served as input networks to predict the remaining protein–protein interactions. (**E**) A contour ternary plot for three hyperparameters and the Matthew correlation coefficient (MCC) on the HI-union dataset is presented. Three hyperparameters were tuned: hidden layer size 'S', number of hidden layers 'H', and dropout probability 'D'. Higher MCC values are indicated by lighter, golden colours, signifying a better model performance on the unseen 25% of genes and all their pairs in the testing set. The best model, with a dropout of 40%, 2 layers, and 60 units, is highlighted in black with an MCC score of 89%. (**F**) UMAP coordinates (identical to those in Fig. 1A–D) are coloured based on pathways from the Kyoto Encyclopedia of Genes and Genomes^61^ (KEGG): 'calcium pathway' with 130 genes, 'Gonadotropin-releasing hormone pathway' (GnRH) (n = 90), 'insulin pathway' (n = 75), Peroxisome proliferator-activated receptors (PPAR) pathway (n = 67), 'transforming growth factor-beta (TGFB) pathway' (n = 85), and 'Toll-like receptor pathway' (n = 49).

To assess the models' capacity to accurately predict PPIs that were eventually discovered, new data was treated as negative observations for older models. Predictions were evaluated both contemporaneously and prospectively (refer to “Methods”). The prospective analysis demonstrated a consistent decline in false positives across each prospective dataset (Fig. 3A), accompanied by an increase in precision (Fig. 3B). The disparity in false positives between consecutive datasets signifies the number of PPIs that could have been prioritised 6–9 years ahead of time (Fig. 3A), with the correctly predicted PPIs ranging between 500 and 2000 in some instances.

True positives that were ultimately discovered in later years, such as 2014 and 2020, were considered as negative samples in the 2005 training set. However, they were still prioritised owing to the positive-unlabeled learning strategy (refer to “Methods”) (Fig. 3A,B). For instance, the majority of HI-union positive but HuRI negative protein interaction pairs were assigned probability scores (HuRI(−) HI-union(+) red histogram in Fig. 3C). These elevated scores correspond to the reduction in false positives for the HuRI model in Fig. 3A and the increased precision for the same model in Fig. 1B.

The PWAS approach outperformed the L3 method from Kovacs et al.^18^ in terms of precision-recall curve at various thresholds for both training and testing sets (Fig. 3D). Despite slight overfitting in the training set (Fig. 3D), the models were able to generalise to previously unseen proteins in the test set. Sixty multilayer perceptron (MLP) models were trained using three different hyperparameters: number of hidden layers, hidden layer size, and dropout percentage (refer to “Methods”) (one dot per model in contour plot of Fig. 3E). To accurately classify PPIs (Matthews correlation coefficient or MCC > 0.8) on the test set, the models required a relatively large hidden layer size (> 10) (Fig. 3E). The best-performing model was D40-N2-S60 (black text in Fig. 3E) with 40% dropout, two hidden layers, and 60 units on the HI-union dataset.

The MLP models using embedding features were able to accurately predict PPIs because the embeddings encode information about the biochemical pathways involving the respective genes (Fig. 3F). For example, members of the peroxisome proliferator-activated receptors (PPAR) pathway, which includes 67 genes (such as PPARA, PPARD, PPARG, RCRA, RXRG in Fig. 3F) from the Kyoto Encyclopedia of Genes and Genomes^61^ (KEGG), clustered together with a mean cosine distance of 0.32, compared to 0.14 for any-to-any gene (Fig. 3F). Similarly, members of the JAK-signal transducer and activator of transcription (STAT) pathway, comprising 148 genes (such as JAK1, JAK2, STAT6, IL4R, IL17RA in Fig. 3F) from KEGG^61^, also clustered together, exhibiting a mean cosine distance of 0.31 compared to 0.14 for any-to-any gene (Fig. 3F).

The MLP model trained with embeddings not only outperformed the L3 method from Kovacs et al.^18^ (Fig. 3D) but also demonstrated the potential to prioritise PPIs for low-throughput experimental validation. Overall, our findings reveal that language models can be harnessed to effectively predict and prioritise PPIs before they are experimentally validated and published, thereby accelerating the discovery of new molecular targets for human diseases.

### Prioritising novel mechanisms of action of drugs

**Figure 4:**
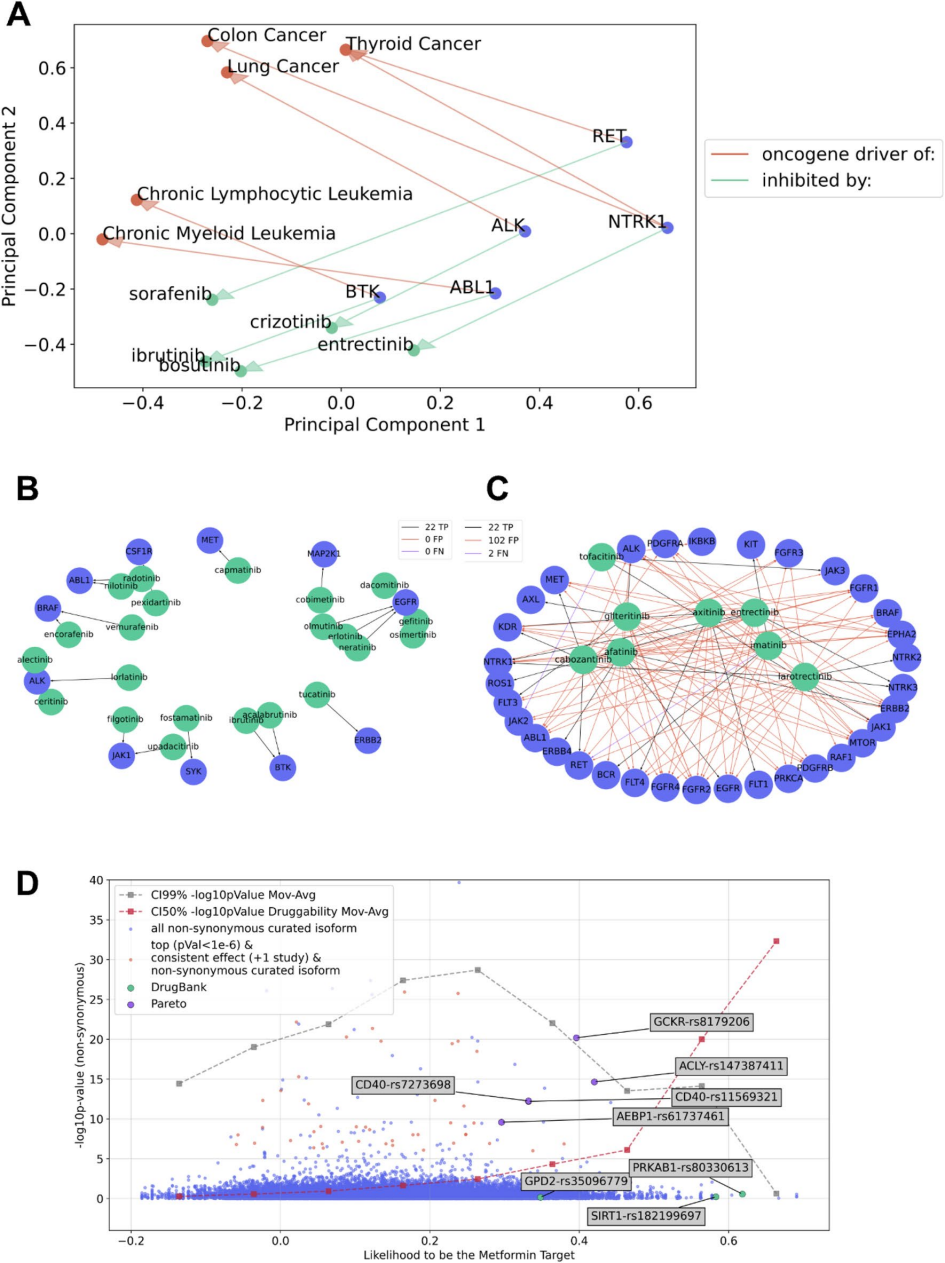
(**A**) Latent relationships learnt between kinase inhibitors, oncogene drivers and cancers. The embeddings for cancer indications (red), oncogenic tyrosine kinases (blue) and kinase inhibitors (green) were projected onto two dimensions using Principal Component Analysis (PCA). There are consistent vector operations between words representing ‘kinase inhibitor of’ and ‘oncogenic gain of function in cancer’. (**B**,**C**) Predicted links between kinase and kinase inhibitors. The predictions were obtained from the best model called H100-N2-D0.2. Network of kinases (blue), kinase inhibitors (green) and their links (true positives) and link predictions: true positives in black, false negatives in purple where any in Fig. 2C and false positives in red where any. The model did not yield any false positives or false negatives for the kinase inhibitors in Fig. 2B. All DrugBank positive links are plotted, and the predicted links with probabilities higher than 0.65. Node size is proportional to the out-degree of each node. The width of the links is proportional to the predicted probability. (**B**) Subplot plots specific drugs with a maximum of two targets. There are no false positives or false negatives. (**C**) Subplot plots promiscuous drugs. There are 102 false positives among the drugs and kinases plotted in the graph. (**D**) Target prioritisation plot for metformin. Scatter plot for the likelihood (x-axis) to be the metformin target and the − log_10_ p-values (y-axis) from the meta-GWAS for non-synonymous variants. The putative targets of metformin SIRT1, AMPK subunit and the mitochondrial glycerophosphate dehydrogenase (GPD2) are in green. Non-synonymous variants with p-values < 10^–6^ and two studies with the same direction of the allele effect are coloured in red. The non-synonymous variants with high likelihoods and genome-wide significance (p-value < 10e−8) are in purple. Two lines were calculated with a range interval moving average from left to right. The 99% confidence interval p-value significance from the GWAS meta-analysis is plotted in grey. The moving average for druggability, as defined by Berenstein et al.^68^, is plotted in red.

Language models, akin to gene-disease associations (Fig. 4A kinase to cancer indication), can also uncover latent associations between proteins and drug-like compounds that modulate their activity (Fig. 4B kinase to kinase inhibitor). A principal component analysis demonstrates a consistent vector operation: 'kinase inhibitor' + 'kinase inhibitor of' ≈ 'kinase' and 'kinase' + 'gain of function in' ≈ 'cancer' (Fig. 4A).

We trained multiple Scikit Learn and TensorFlow models (Table 5) using gene and compound word vectors as input, with positive samples from DrugBank and random negative pairs (see “Methods”). The gene and compound vectors were concatenated to generate a single fingerprint for each target-compound pair. These models were employed to weight features and produce a single score for each gene-compound pair, estimating the likelihood of compound modulation of the protein encoded by the gene. The H100-N2-D0.2 model, a multilayer perceptron classifier with 100 hidden neurons (H100), 2 hidden layers (N2), and 0.2 dropout rate (D0.2), delivered the best performance.

The model's precision was relatively low, with only 17% of true positives correctly predicted with high probability across all positives (Table 5). However, when grouped by drug-like compound specificity (Table 2), precision increased from 17% (Table 5) to 86.1% for compounds with 2 targets reported in DrugBank and 95% for compounds that act on one human protein target (Table 2, “Pharmacologically active (1 Target)”) while still being low for unspecific compounds with more than 2 targets with a 12.7% precision (Table 2).

**Table 2:** Classification accuracies grouped by promiscuous drugs. Classification statistics for predicting links from compounds whose pharmacological mechanism of action is known or at least reported in DrugBank and their protein targets. The results presented in this table are only for the testing set unseen during training. The table is grouped by the number of reported protein targets in DrugBank (more than 2, 2 or 1 target).

Metric	No active (Num. targets > 2)	Pharmacologically active (Num. targets > 2)	No active (2 targets)	Pharmacologically active (2 targets)	No active (1 target)	Pharmacologically active (1 target)
Accuracy score	0.835	0.835	0.869	0.869	0.863	0.863
F1 score	0.907	0.221	0.896	0.822	0.871	0.856
Precision score	0.995	0.127	0.873	0.861	0.799	0.950
Recall score	0.834	0.856	0.920	0.786	0.956	0.778

The H100-N2-D0.2 model demonstrated low specificity (12%) for promiscuous compounds acting on three or more targets (Table 2), such as unspecific kinase inhibitors targeting multiple kinases (Fig. 4C). The H100-N2-D0.2 multilayer perceptron classifier accurately assigned high scores to selective kinase inhibitor targets, including vemurafenib and encorafenib for BRAF, osimertinib, erlotinib, gefitinib, and neratinib for EGFR, and ibrutinib and acalabrutinib for BTK (Fig. 4B).

As demonstrated in Table 2, the language models could prioritise known mechanisms of action for drug-like compounds. Therefore, we tested these models on drugs with unresolved targets, such as metformin, a widely-used medication for improving glucose metabolism and alleviating diabetes-related complications. Although several metformin targets have been identified, including AMPK, the mitochondrial respiratory chain complex 1, and mitochondrial GPD2, its precise mechanism of action remains elusive^62^.

We examined the genome-wide target ranking for metformin in comparison to genome-wide association data (refer to “Methods”) for glycemic response (Fig. 4D), focusing on non-synonymous variants that result in amino acid changes. The putative metformin targets, such as PRKAB1 (a component of the AMPK complex), SIRT1, and GPD2, ranked highly (green dots in Fig. 4D). The language model assigned higher scores to small molecule tractable targets (CI50% tractability red line in Fig. 4D, see “Methods”). However, there was a depletion of p-values from the GWAS meta-analysis (CI99% p-value grey line in Fig. 4D), and none of the putative targets exhibited significant non-synonymous variants.

Five variants in four genes (Table 3) met several criteria: non-synonymous, p-value lower than 10e−9, consistency in the beta coefficient across two studies, and a high likelihood (> 0.3) of being a metformin target. The highest-ranked variant, rs8179206 (Glu77Gly) in the GCKR gene, has been associated with elevated triglycerides in the blood^63^ and obesity^64^. However, no publications have linked the genetic variants rs7273698 and rs7273698 in CD40, rs61737461 in AEBP1, and rs147387411 in ACLY to metformin (Table 3, Fig. 4D).

**Table 3:** Top-ranking genes. Gene symbol, allele change location in the Genome Reference Contig GRCh37, amino acid replacement, reference single nucleotide polymorphism (SNP) identifier, beta coefficient, − log10 of the p-value, the number of independent studies from the meta GWAS, number of samples as the sum of each study, and the direction of the effects: the sign of the beta coefficient (Minus sign for negative, plus sign for positive coefficients, interrogation sign for variants not measured and coefficients not calculated). Genes in this table were non-synonymous, had a p-value lower than 10e−9, had two studies with the same direction in the beta coefficient, and had a high likelihood of being the target of metformin according to the language model.

Gene	Variant	Allele frequency	Amino acid change	Beta coefficient	− log10 p-value	Number meta GWAS	Samples	Direction effects
ACLY	rs147387411	< 0.001	Arg582Gln	-3.438	14.644	2	7048	??????–
AEBP1	rs61737461	< 0.001	Ile444Leu	-1.595	9.589	2	7048	??????–
CD40	rs11569321	< 0.001	Ser124Leu	-1.673	12.21	2	7048	??????–
CD40	rs7273698	< 0.001	Phe150Phe	-1.675	12.261	2	7048	??????–
GCKR	rs8179206	< 0.001	Glu77Gly	-2.945	20.18	2	7048	??????–

ATP citrate lyase (ACLY) is involved in converting citrate and coenzyme A into acetyl-Coenzyme A and oxaloacetate^65^ across various high-fat-producing tissues, including the liver, adipocytes, and pancreatic beta cells68. Acetyl-CoA contributes to lipogenesis and cholesterogenesis pathways^65^. ACLY is a promising therapeutic target for cholesterol reduction and protection against atherosclerosis^66^. The ubiquitination and subsequent degradation of ACLY shifts cell metabolism from synthesis to fatty acid oxidation^66^, correlating with metformin's activation of AMPK, stimulation of fatty acid oxidation, inhibition of cholesterol and triglyceride synthesis^67^, and increased glucose uptake and insulin sensitivity in skeletal muscle^67^.

The Arg592Gln (rs147387411) mutation is located near the coenzyme A binding domain but not directly within it. Acetylation at three lysine residues (K540, K546, and K554) by lysine acetyltransferase 2B (KAT2B) has been shown to increase ACLY stability by blocking its ubiquitylation and promoting de-novo lipid synthesis^67^. ACLY is deacetylated and inhibited by SIRT2, but not SIRT1^67^, a putative metformin target. The Arg592Gln ACLY mutant may exhibit altered acetylation, ubiquitination, or phosphorylation upon metformin treatment. However, additional experimental work is necessary to confirm its connection with metformin.

## Discussion

The Publication-Wide Association Study (PWAS) leverages language models to analyse 19.5 million publications, prioritising previously unpublished hypotheses across various contexts: gene-disease associations, target-disease clinical trials, protein–protein interactions, and unexplored yet plausible drug mechanisms of action. The study demonstrates that word embeddings, or vector representations of words, can effectively encode complex biomedical knowledge from published literature without requiring a priori biomedical knowledge. These embeddings capture intricate biomedical concepts, such as drug target classes, tractability, disease phenotype involvement, and protein–protein interactions. Furthermore, language models have successfully ranked novel hypotheses years before their publication in top-tier journals, suggesting that the foundations of future discoveries are already embedded within existing publications to some extent.

To the best of our knowledge, this is the first attempt to utilise language models for prioritising drug discovery hypotheses prior to their publication. Language-based inference methods may serve as a promising new research field at the intersection of natural language processing and target identification, propelling language models beyond their traditional use in named entity recognition and normalisation tasks to harness the wealth of associations present in biomedical literature. The various tasks for language models will be discussed in the following sections.

### Target prioritisation

The vector representations of genes contain information about small molecule and antibody druggability, disease association, therapeutic target family, pathways and clinical precedence that can be generalised to unseen genes (Fig. 2). These embeddings contained more information than manually engineered features by biology experts^13^ (Table 1). Around 50% of the top-ranked genes ended up being published as associated with the disease within 10 years, a similar percentage to the results from Tshitoyan et al.^69^. Furthermore, some relevant gene-disease associations validated could have been prioritised one to several years before their publication. The PWAS method outperformed the Paliwal et al.^12^ method at prioritising clinical trial targets for 200 diseases in the test set. However, language models cannot make predictions for genes or diseases that are not in the training data. Genome-wide association studies have suggested for the first time disease associations for novel genes, not previously published (not in the training data). These associations cannot be prioritised with language models unless scientists write paragraphs describing the gene function based on experimental work. Furthermore, PWAS is another association study far from prioritising causal genes. Paradigm shifts in molecular biology and spurious associations between genes and diseases will bias the models. Furthermore, scientific literature is noisy and often contradictory. Despite these problems, the PWAS could prioritise some targets several years before their first publication and before their first clinical trial. Future work will require training more complex transformer models at hypothesis generation and question answering.

### Protein–protein interactions

In this study, the PWAS exhibited enhanced performance in comparison to L3^18^, as depicted in Fig. 4E. The ability of language models to prioritise protein–protein interactions (PPIs) in advance offers a promising opportunity to supplement experimental approaches for completing the human interactome. However, there are limitations to consider. Unlike other published methods^23–25^, the predictions in this study do not identify the interface residues essential for the interaction. Additionally, the current work heavily depends on high-throughput yeast-two-hybrid (Y2H) experiments, known for limited sensitivity and specificity^58–60^. Y2H experiments face a significant drawback, as they cannot capture PPIs requiring post-translational processing, a common occurrence in humans, and only focus on a single isoform per gene, excluding alternative splicing variants that may have crucial roles in protein function and interactions.

Moreover, this study did not incorporate several independent and competing PPI databases, such as IntAct^70^ or STRING^71^. These resources offer valuable information on experimentally validated and predicted PPIs, and their inclusion could potentially enhance the comprehensiveness and accuracy of human interactome mapping.

### Drug target interactions

Language models demonstrated the ability to learn known target-drug relationships for specific compounds effectively. Notably, the putative targets of metformin prioritised by these models and the genome-wide ranking generated strongly correlated with independent small molecule tractability scores. It is hypothesised that the Arg592Gln ACLY mutant might undergo differential acetylation, ubiquitination, or phosphorylation in response to metformin treatment. However, this hypothesis necessitates further experimental validation to establish its veracity conclusively.

### Limitations

Despite the vast potential of language models trained on biomedical literature, it is crucial to acknowledge their inherent limitations. One primary concern is the presence of biased, erroneous, irrelevant, and weak studies within the training data. These suboptimal sources may introduce noise and inaccuracies, thereby corrupting the model's knowledge base and impairing its ability to make accurate predictions or generate reliable insights. Consequently, the conclusions drawn from such models could be compromised, leading to misguided hypotheses and, ultimately, impeding scientific progress. Therefore, it is essential to exercise caution when interpreting the outputs of language models and to validate their findings through independent experimental approaches to ensure the veracity and robustness of the generated results.

Named entity recognition (NER) errors can greatly affect the quality and dependability of a publication-wide association study (PWAS) in target-disease prioritisation. These inaccuracies may result in false or missed associations, misrepresented relationships, and diminished trust in the model outputs. While advanced NLP techniques and transformer models, such as BioBERT or GPT-based architectures, may offer improvements, they still face challenges in ranking extensive lists, like 20,000 human genes. As these models tokenise input sequences into smaller units, gene names or symbols can be divided into multiple tokens, complicating the generation of a precise genome-wide ranking approach. Future research will need to be made to replicate the PWAS work with transformer models and evaluate them in a retrospective analysis to prove they can prioritise novel preclinical targets that are eventually successful in the clinic.

A further limitation concerning entity recognition lies in the representation of diseases as singular entities with a unified vector representation. However, diseases are heterogeneous, and gene alterations contribute variably to the onset and progression of the condition. Certain genes may serve as potential targets for the initial manifestation, while others play a role in exacerbating and aggravating the disease's progression. This study generates a single genome-wide ranking for each disease, regardless of its state or trajectory. Nonetheless, some disease stages are represented by distinct ontology terms within the MeSH ontology, such as the progression from NAFLD to non-alcoholic steatohepatitis and ultimately to hepatocellular carcinoma where the rankings will be slightly different. Moreover, target prioritisation encompasses a multi-objective ranking approach where the target needs to be safe, tractable and associated with the disease. Incorporating additional objectives or keywords could help prioritise targets for particular phenotypes of the disease. For example, combining associations to DNA-damage repair keywords with safety and tractability keywords along with cancer types to bias the initial ranking, similar to how one would pose a question with more detail to a transformer model.

## Conclusion

The findings of this study indicate that to some extent, latent information regarding future discoveries is embedded within past scientific publications. Furthermore, language models present a compelling method for prioritising unverified hypotheses derived from the continuously growing corpus of biomedical literature. To our knowledge, this work represents the first literature-wide association study that ranks therapeutic drug hypotheses for subsequent validation utilising language models trained on historical corpora.

Efficient assimilation of existing knowledge, identification of promising research directions, and avoidance of redundant efforts are all vital components for scientific progress and hypothesis generation in target analysis. However, with the ever-increasing volume of biomedical literature, this task becomes progressively more challenging, if not unfeasible, for individual researchers.

Our research has the potential to contribute to a novel paradigm wherein the vast amount of information contained in scientific publications becomes readily accessible to individual target analysts, thereby fostering machine-assisted breakthroughs in the identification of innovative targets. The Publication-Wide Association Study (PWAS) represents a scalable system that facilitates the prioritisation of under-explored targets, expediting early-stage drug target selection irrespective of the specific disease of interest. This method holds the potential to radically transform the way researchers navigate and leverage the extensive knowledge base within the biomedical field.

## Methods

### Training corpus, vocabularies and preprocessing

The input data for this study comprised roughly 19.5 million English abstracts sourced from PubMed, which included mentions of any human gene or disease synonyms in their titles or abstracts. Abstracts classified as 'Commentary', 'Correction', and 'Corrigendum' in PubMed were excluded. In this study, we utilised a comprehensive set of 4,819 disease terms from the Medical Subject Headings (MeSH) ontology, which encompasses a diverse range of diseases, phenotypes, and symptoms. MeSH, developed by the National Library of Medicine, is a controlled vocabulary used for indexing and cataloguing biomedical literature in the MEDLINE/PubMed database. Furthermore, regarding the gene vocabulary, we have taken into account 19,229 human protein-coding genes.

The TrendyGenes^72^ pipeline was employed to identify biomedical entities, specifically genes and diseases, within these abstracts. Standardised identifiers were utilised to normalise genes and diseases, a process that involves canonicalising all concept synonyms. For instance, 'Her2', 'ERBB2', and 'Neu' were normalised into their Ensembl identifiers, while 'non-insulin-dependent diabetes', 'diabetes mellitus, type II', and 'T2DM' were normalised to the corresponding Medical Subject Headings (MeSH) identifier for type 2 diabetes.

Normalisation was implemented for several reasons. Firstly, it reduces the vocabulary size. Secondly, it accounts for different research groups using distinct gene synonyms in various research contexts (e.g., genetics using official gene symbols versus biochemistry articles). Canonicalization consolidates information into a single token, enabling the generation of a genome-wide ranking of 20,000 human protein-coding genes, rather than n_synonyms multiplied by the number of genes. Normalising biomedical entities led to a fivefold reduction in gene synonym mentions, from 102,719 to 19,229 human protein-coding gene identifiers. Similarly, disease synonym mentions were reduced tenfold, from 53,317 to 4819 human disease identifiers. Proper corpus preprocessing significantly enhances the performance of language models^69^.

The vocabulary for word2vec models included all words that appeared more than ten times and normalised gene and disease identifiers, regardless of the number of mentions. As a result, historical models featured different vocabularies. If a gene is never mentioned in the training corpus, it is excluded from the language model's vocabulary. For instance, 13,676 distinct human genes were mentioned in the training corpus up to 2005, while 16,533 different human genes were mentioned until the present, accounting for 86% of the total 19,229 human protein-coding genes. Normalised gene and disease mentions refer to the mapping of any textual references to a unique identifier from the HUGO or the Medical Subheadings Ontology (MeSH).

Leading statements such as 'Background:', 'Abstract:', and 'Introduction:' were removed. Phrases were generated using a minimum phrase count of 10, normalised mutual information score greater than 0.7, and phrase depth up to three times. This phrasing process was repeated thrice, allowing the generation of up to 8-g. For example, the phrase 'g protein coupled receptor' appeared more than ten times in the corpus and had a normalised mutual information score greater than 0.7, which was then phrased into 'g_protein_coupled_receptor'. The text was converted to lowercase and deaccented. Floating decimals and percentage numbers were replaced with the word '< number >' using regular expressions to decrease vocabulary size. Stop words were retained as they constituted approximately 100 tokens within the half-million-token vocabulary.

### Language models

We employed the Word2Vec algorithm from the Gensim Python library for our analysis. A range of hyperparameter combinations were explored to identify the optimal configuration for capturing gene target to disease indication analogies (Table 4). We selected a window size of 10, which corresponds to the median distance (in words) between diseases and genes in PubMed. Based on the work by Yin and Shen^73^, an embedding size of 255 minimised the Pairwise Inner Product loss, though we also conducted a grid search with sizes of 128, 256, and 512. The remaining hyperparameters were set to their default values.

**Table 4:** Hyperparameter grid search for various Word2Vec models. The hyperparameters explored include the Word2Vec model task, either contextual bag-of-words (CBOW) or skip-gram (SG); the size of the embedding vectors, represented by the number of variables in the word vectors as multiples of 2 (128, 256, and 512); and the window distance, which is the number of tokens (words or phrases) between the central pivot word and the surrounding words that the algorithm attempts to predict. Table entries indicate the vocabulary ranking of the correct answer for analogies of the form: target A is to disease A as target B is to disease B, as originally demonstrated by Mikolov in their publication^26^. The best score is highlighted in bold and with an asterisk.

Task	Embedding size	Window Distance		
		5	10	15
CBOW	128	1052	856	899
CBOW	256	987	771*	813
CBOW	512	1123	903	964
SG	128	954	899	904
SG	256	943	792	891
SG	512	923	851	903

During the 50 training epochs, the learning rate was linearly reduced from 0.01 to 0.0001. We experimented with context or distance window sizes of 5, 10, and 15 tokens. Subsampling was performed with a 10^−4^ threshold, targeting approximately the 400 most frequent tokens in the vocabulary. Both Word2Vec variants, skip-gram and continuous bag of words (CBOW), were considered. The chosen model, which maximise performance on a curated dataset of target-to-disease analogies (Table 4), was the CBOW model with an embedding size of 256 and a window distance of 10.

### Target-disease prioritisation

Literature associations between genes and diseases were obtained from the TrendyGenes pipeline^72^. The clinical stages for the target-disease indications were gathered from the manual curation of Pharmaprojects^54^ and Open Targets^13^ datasets. The ‘chembl’ score from Open Targets^13^ was recalculated after the integration: a score from 0 to 1 depending on the clinical phase of a target-disease pair: 0.1 for phase 1, 0.2 for phase 2, 0.7 for phase 3 and 1.0 if there is an approved and launched drug. This step scored each target-disease association depending on the clinical trial stage. Gene expression data in non-small cell lung cancer in Fig. 1I was obtained from The Cancer Genome Atlas^74^. The threshold was a transcript per million of 0.1 from the lung adenocarcinoma dataset (TCGA-LUAD).

For Fig. 1J,K, multiple feed-forward neural networks with different amounts of dropout, hidden layer sizes and the number of layers were used to regress the ‘chembl’ score from 2016 for Fig. 1J,K. Three hyperparameters were tuned: the number of hidden layers (1 or 2), the dropout (0, 10, 20, 30 or 40%) and the size of the hidden layers (1, 5, 10, 20, 50, 100). The model with the highest validation accuracy on the future (test) set was selected. Embeddings from December 2015 were used as covariate features to regress the ‘chembl’ scores, giving higher scores to approved drugs than to target-disease pairs in phase 1. We use data from 2016 to demonstrate the ability to prioritise prospective associations until 2022. The training and testing data were split according to Paliwal et al.^12^ 2020 publication: 200 diseases and all their targets were in the testing set, and the remaining disease and their targets were part of the training set. In the supplementary methodology section, Paliwal et al.^12^ provided supplementary information for only 40 out of the 200 diseases. The remaining 160 diseases were randomly selected to encompass a variety of disease types, including metabolic, immune, neurological, and oncological indications. To achieve this, diseases were first categorised into four groups (metabolic, immune, neurological, and oncology). Only diseases with at least three targets in Informa were considered for inclusion in the testing set, while over-specific diseases situated at the leaf nodes of the ontology tree were excluded. Utilising a uniform distribution, 160 diseases were then randomly selected from each category, 40 diseases from each therapeutic area. All remaining diseases with at least one target were allocated to the training and validation sets.

For Fig. 1L, genome-wide summary statistics to run the MAGENTA pipeline^52^ were obtained from Wang et al. 2021 data^53^. MAGENTA ran with default parameters with the summary statistics and all data in the genome build Genome Reference Consortium 37 (GRCh37). To run the Priority index^21^ (Pi in green Fig. 1L), we used a genome-wide summary statistics pipeline from Open Targets Genetics^13^. A total of 15,901 genes are prioritised, based on 6645 single nucleotide polymorphisms (SNPs) scored positively (including 644 'Lead' and 6001 'Linkage Disequilibrium' genes); 714 nearby genes within 50,000 base pairs genomic distance window of 6129 SNPs 2045 expression Quantitative Trait Loci (eQTL) genes with expression modulated by 4677 SNPs; 912 HiC genes physically interacted with 4033 SNP; 2900 genes defined as seeds from 6645 SNPs; randomly walk the network (15,901 nodes and 318,866 edges from the STRING database^71^) starting from 2900 seed genes with a restarting probability of 0.75. The Open Targets dataset only gives a score of 0 and 1 to targets with evidence for diseases. To generate a genome-wide ranking to calculate the z-scores, the remaining non-associated targets were given a random score (uniform distribution) between the minimum association score for the disease and zero. Z-scores were calculated with the quantile normalisation function in Scikit Learn.

The list of therapeutic targets in this section was downloaded from Pharmaprojects and included all therapeutic targets for systemic lupus erythematosus that had an active program in the following stages: Phase I Clinical Trial, Phase II Clinical Trial, Phase III Clinical Trial, Registered, and Launched. This included the following gene symbols: BTK, BTLA, CD200, CD200R1, CD22, CD28, CD38, CD40, CD40LG, CD6, CD79B, CLEC4C, CNR2, CRBN, CXCR5, FCGR2B, FCGRT, FKBP1A, ICOSLG, IFNA1, IFNAR1, IKBKE, IL12B, IL1RL2, IL2, IL21, IL23R, IL2RA, IL2RB, IL2RG, IRAK1, IRAK4, JAK1, JAK3, LANCL2, LGALS1, LGALS3, LILRA4, MALT1, MASP2, MC2R, MIF, MS4A1, NLRP3, NR3C1, PIK3CB, S1PR1, SIK2, SLC15A4, SNRNP70, SOCS1, STING1, SYK, TBK1, TLR7, TLR8, TNFRSF13B, TNFRSF13C, TNFRSF4, TNFSF13, TNFSF13B, TYK2, XPO1.

### Therapeutic target representation

The clinical stages for the targets were gathered from Pharmaprojects^54^ and Open Targets^13^ datasets. The therapeutic target classes and the kinase families were downloaded from the Guide to Pharmacology database^57^. The Uniform Manifold Approximation and Projection (UMAP) low dimensional representation was achieved using the umap-learn package in Python with the following hyperparameters: 15 neighbours, cosine similarity as the metric, 1000 epochs, a repulsion strength of 12 and local connectivity of 3 neighbours. To measure the consistency of the clusters in Fig. 1A, the differences in the cosine distances were compared. The cosine distance represents the angle between two vectors. The more similar the vectors, the closer the angle to 1. The intercluster cosine distance was significant for all gene families with the following P-values using a t-test with unequal variance and a nonparametric Mann–Whitney U test: 3.03e−251 and 7.49e−249 for enzymes, 3.17e−321 and 0.0 for G-protein coupled receptors, 2.45e−304 and 6.96e−166 for kinases, 4.38e−124 and 3.51e−46 for voltage-gated ion channels (see “Methods”).

We adopted the same hyperparameters as Ferrero et al. in 2017^9^ for the multilayer perceptron, a feed-forward neural network with a single layer and a balanced function implemented in SciKit Learn. The solver was Adam, and the network stopped if the performance did not improve for two epochs in a validation set. The input data was the gene embeddings from the language model from December 2016 to directly compare to Open Targets features at the time. The metrics displayed in Table 1 correspond to an independent, balanced test set. The same model architecture was used to classify a gene into a tractable target by monoclonal antibodies and small molecules in Fig. 1G,H. The data for the labels for whether a target is tractable with small molecules or antibodies was gathered from manual curation of Pharmaprojects^54^ and Open Targets. The datasets in this section were randomly split into training and test sets, containing 80 and 20% of the observations (similar to Ferrero et al.^9^), respectively.

### Protein–protein interactions

**Figure 5:**
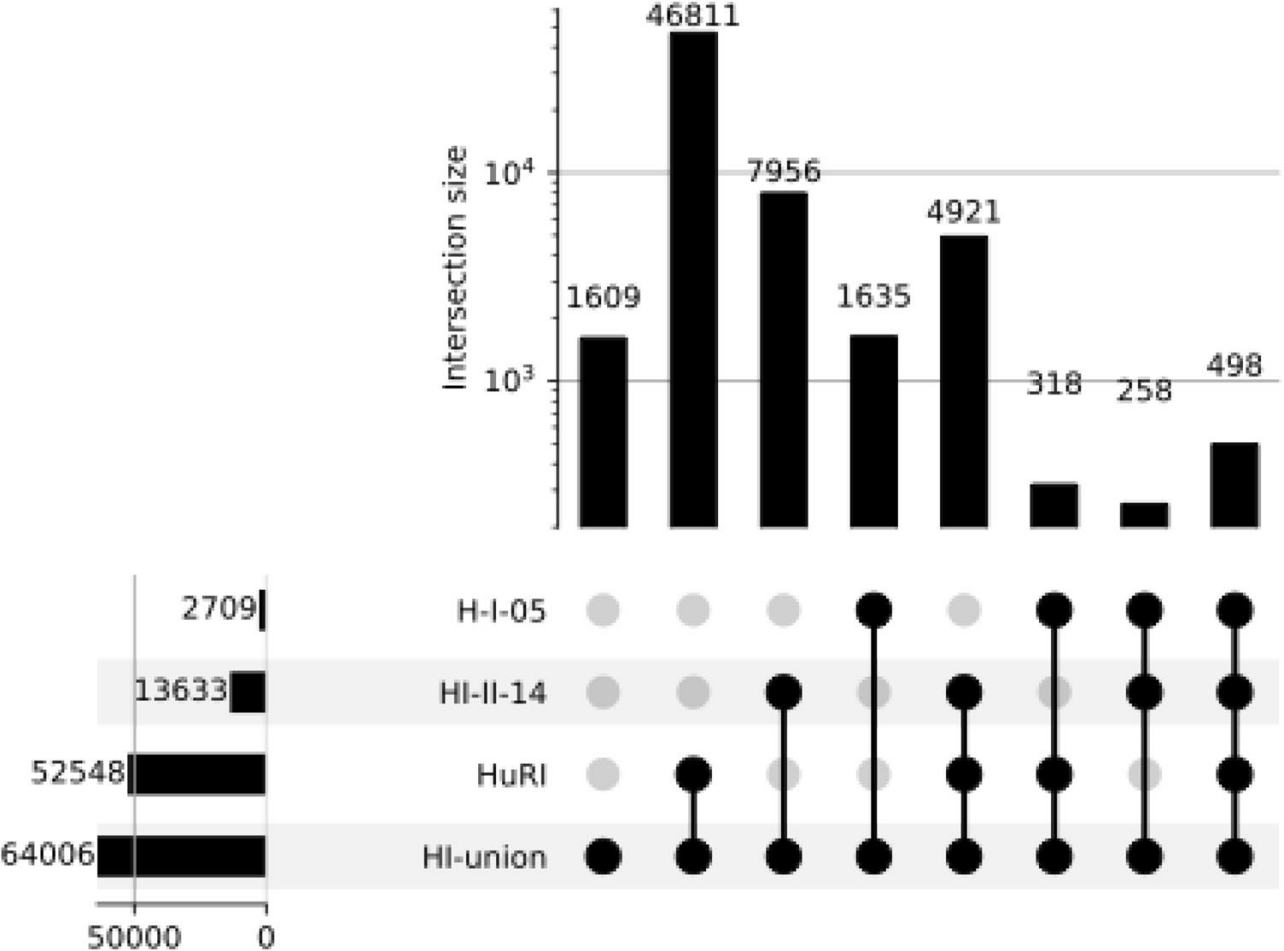
Upset plot of the Human Interactome. The upset plot shows the overall number of unique protein–protein interactions and the size of the intersecting pairs among the datasets: HuRI published in Nature, 2020^58^; HI-union adds literature curated interactions to HuRI; HI-II-14 published in Cell, 2014^59^; and H-I-05 published in Nature, 2005^60^. Each count corresponds to a unique, alphabetically sorted protein–protein pair, this is, 'ZNF44-A1BG' =  >'A1BG-ZNF44' based on the approved gene symbol. Self-interactions were discarded like auto-phosphorylations in tyrosine receptor kinases. Every possible intersection of protein pairs is represented in the histograms. The total number of protein–protein interactions from each historical dataset is displayed in the histogram on the left.

We used all historical versions of the human interactome^58–60^, accounting for 64,000 human protein–protein interactions in its latest release. Self-interactions were discarded like self-phosphorylations in tyrosine kinase receptors. Protein interaction pairs were augmented by reversing the gene order (e.g. 'A1BG-ZNF44' =  > 'ZNF44-A1BG'). These interactions were positive examples. There was no *bona fide* negative set. Therefore, all combinations of the top 10% of proteins with most interactions were negative examples unless they intersected with the positive set. For example, even though the genes DVL2 and TRAF2 had 36 and 69 interaction pairs, the DVL2-TRAF2 pair was excluded from the negative pair set because it was in the H-I-05 positive pair set. However, the pair DVL2-NIF3L1, with 36 and 31 interactions respectively in H-I-05, was included in the set of negative pairs. An upset plot for the different protein–protein interactions coming from each of the historical datasets can be seen in Fig. 5, where 498 unique protein–protein interactions were common across all datasets and HI-union contained a total of 64,006 protein–protein interactions of which 46,811 were intersecting with HuRI.

To test the ability of the models to eventually recover true protein–protein interactions, the newly positive interactions in 2020 were regarded as negative examples to the 2014 and 2005 models. For example, the CCHCR1-ZWINT interaction introduced in HuRI was a positive example for the 2020 experiment but negative for the 2014 and 2005 experiments. The idea was to test whether a positive-unlabelled strategy^75^ could prioritise future protein interaction pairs even if they were presented as negatives. The features were the concatenation of the two embeddings for the proteins in the pair. Language models were checkpointed each December. Embeddings trained until December 2005 were the features for the model trained on H-I-05 examples, embeddings from December 2014 embeddings to train with HI-II-14 positive examples and embeddings from December 2020 embeddings with positive examples from HuRI and HI-union.

The split into training, validation and test sets was done in two ways. There was a protein-based split for testing. A random split of 1:3 for the test and train sets at the protein but not the pair level. 25% of genes and all their partners are just on the test set and never seen during the training. Also, there was a time-based split for validation. The predictions were also evaluated prospectively in Fig. 3A–C. Language models were trained up to different dates in the past. Neural network models with past embeddings were trained with old protein interaction pairs and evaluated on newer datasets. For example, the H-I-05 multi-layer perceptron was trained with 2005 embeddings and H-I-05 examples. This H-I-05 multi-layer perceptron was evaluated on H-I-05 interaction pairs and prospective data: HI-II-14 and HuRI and HI-union (Fig. 3A–C).

A grid search was performed for the hyperparameter tuning. Three hyperparameters were tuned: the number of hidden layers (1 or 2), the dropout (0, 10, 20, 30 or 40%) and the size of the hidden layers (1, 5, 10, 20, 50, 100). This tuning generated 60 different models with different combinations of hyperparameters that resulted in the contour ternary plot in Fig. 3E.

### Prioritising novel mechanisms of action of drugs

The compound-protein interaction data was sourced from DrugBank^76^, comprising over two thousand curated, high-quality binary interactions involving human proteins. Positive examples were derived from this dataset, while negative examples were generated by combining the 400 most frequently occurring proteins with the 400 least specific compounds. These negative examples did not overlap with the positive set. This approach was adopted for three reasons: (i) to improve false association discrimination by incorporating non-specific drugs and targets in the negative sets, enabling models to better differentiate between true and false protein-drug associations so the models do not just predict that staurosporine, sunitinib and sorafenib inhibit all kinases; (ii) to enhance model robustness by introducing difficult negative examples, leading to more robust and generalisable models; and (iii) to ensure rigorous model evaluation by presenting a challenging negative set, characterised by a higher potential for false positives.

Multiple machine learning classifiers were trained to prioritise positive and negative compound-target interactions, including random forest classifiers and logistic regression implemented in Scikit Learn and deep neural networks in TensorFlow. The neural networks have a variable number of layers, dropouts and sizes of their hidden layers. All models have a balanced loss function to account for the imbalance in the dependent variable. All models used are displayed in Table 5.

**Table 5:** Compound-protein model evaluation. Test set results are ranked by Matthews correlation coefficient (MCC). The optimal model, H100-N2-D0.2, utilises a neural network with 100 hidden neurons (H), 2 hidden layers (N) for pairwise interactions of fingerprint word embeddings, and a 0.2 dropout probability (D). The next best model employs a random forest with 100 estimators and Gini entropy for bootstrap selection. All models utilise a balanced loss function to address dependent variable imbalance. The logistic regression model outperforms a basic neural network with a single hidden layer and a size of 1. The highest score is denoted by bold text and an asterisk.

Model	Matthews correlation	Precision	Recall
H100-N2-D0.2*	**0.345**	**0.174**	**0.876**
RFC	0.344	0.261	0.549
H100-N2-D0.0	0.344	0.172	0.882
H100-N3-D0.0	0.343	0.172	0.872
H50-N3-D0.0	0.342	0.175	0.856
H50-N3-D0.2	0.333	0.164	0.878
H50-N2-D0.0	0.325	0.159	0.873
H50-N2-D0.2	0.319	0.153	0.886
H100-N3-D0.2	0.318	0.152	0.888
H100-N1-D0.2	0.308	0.149	0.862
H100-N1-D0.0	0.307	0.147	0.871
H50-N1-D0.0	0.307	0.149	0.859
H50-N1-D0.2	0.307	0.149	0.856
H10-N3-D0.2	0.287	0.144	0.802
H10-N2-D0.0	0.281	0.132	0.853
H10-N2-D0.2	0.273	0.131	0.83
H10-N1-D0.0	0.269	0.128	0.83
H10-N3-D0.0	0.265	0.123	0.847
H10-N1-D0.2	0.265	0.129	0.805
H1-N1-D0.2	0.257	0.13	0.764
Logistic Regression	0.249	0.117	0.821
H1-N1-D0.0	0.244	0.121	0.77
H1-N2-D0.0	0.177	0.086	0.756
H1-N2-D0.2	0.168	0.079	0.795
H1-N3-D0.2	0	0	0
H1-N3-D0.0	0	0	0

We used the latest release of the InterPro database^77^ for protein-motif links. InterPro classifies protein amino acid sequences into families and predicts the presence of functionally essential domains and sites. For the small molecule tractability, we followed the implementation of Berenstein et al.^68^, where each protein target had a tractability score. Each protein had the maximum tractability score from the p-value of a Fisher’s exact test for their constituent InterPro^77^ motifs. InterPro motifs were associated with compounds through a motif-protein-compound graph. This approach gives non-trivial scores to proteins with druggable motifs if another protein with the same motif has been drugged.

The genome-wide association study in Fig. 4D used the combined meta-analysis for glycemic response to metformin^78^. This dataset contains 1024 Scottish individuals with type 2 diabetes and two replication cohorts: 1113 individuals from the UK Prospective Diabetes Study and 1783 Scottish individuals. MyVariant^79^ was used as a service to query multiple variables regarding each single nucleotide polymorphism, including non-synonymous replacements and their functional consequences.

## Data Availability

The datasets employed and analysed in this study can be obtained from the corresponding author upon a reasonable request. All the datasets mentioned in this work were downloaded on the 28th of February, 2022.
